# Correction: “Prompt Engineering an Informational Chatbot for Education on Mental Health Using a Multiagent Approach for Enhanced Compliance With Prompt Instructions: Algorithm Development and Validation”

**DOI:** 10.2196/75191

**Published:** 2025-04-10

**Authors:** Per Niklas Waaler, Musarrat Hussain, Igor Molchanov, Lars Ailo Bongo, Brita Elvevåg

**Affiliations:** 1 Department of Computer Science UiT The Arctic University of Norway Tromsø Norway; 2 Department of Clinical Medicine UiT The Arctic University of Norway Tromsø Norway

In “Prompt Engineering an Informational Chatbot for Education on Mental Health Using a Multiagent Approach for Enhanced Compliance With Prompt Instructions: Algorithm Development and Validation” (JMIR Res Protoc 2025;4:e69820) the authors noted two errors.

The affiliation of Per Niklas Waaler was changed from:

Department of Computer Science, UiT The Arctic University of Norway, Lund, Sweden

to:

Department of Computer Science, UiT The Arctic University of Norway, Tromsø, Norway

The contact information for the corresponding author was also changed from:

Corresponding Author:

Per Niklas Waaler, MS

Department of Computer Science

UiT The Arctic University of Norway

Backgatan 35, Södra Sandby

Lund, 24731

Sweden

Phone: 46 944 44096

Email: pwa011@uit.no

to:

Corresponding Author:

Per Niklas Waaler, MS

Department of Computer Science

UiT The Arctic University of Norway

Hansine Hansens vei 54

Tromsø, N-9037

Norway

Phone: 47 776 44056

Email: pwa011@uit.no

The correction will appear in the online version of the paper on the JMIR Publications website together with the publication of this correction notice. Because this was made after submission to PubMed, PubMed Central, and other full-text repositories, the corrected article has also been resubmitted to those repositories.

