# Supplementary material for: Prompt Engineering an Informational Chatbot for Education on Mental Health Using a Multiagent Approach for Enhanced Compliance With Prompt Instructions: Algorithm Development and Validation
Source: JMIR AI. 2025 Mar 26;4:e69820. doi: 10.2196/69820 (PMC11982747; doi:10.2196/69820)
Supplement: Multimedia Appendix 1 [file ai_v4i1e69820_app1.doc]

## Overview

We have prompt-engineered a chatbot whose purpose is to convey the contents of a schizophrenia manual in a chatbot environment. To anchor the chatbot's responses in reliable sources, we implement an **Information Retrieval Algorithm** whereby the chatbot retrieves documents from a knowledge database as the need arises. The documents are sections of text, referred to herein as ***sources***, extracted from the GAMIAN manual “Learning to Live with Schizophrenia”. The desired behavior of the chatbot is defined by the instructions in the initial prompt and by the information in the documents that it has access to. Because adherence to instructions tends to weaken over a conversation, we have designed a **Critical Analysis Filter** (CAF) which is designed to ensure that the chatbot’s responses are aligned with the sources and the instructions of the chatbot. More specifically, a team of prompted LLM agents, referred to herein as ***overseers***, monitor and process the chatbot's response to ensure that the chatbot's response aligns with the chatbot’s instructions and sources. To ensure that the chatbot’s responses are consistent with the information in its knowledge base (i.e., the schizophrenia manual) and stays consistent with its instructions, we require that the chatbot starts each message with a ***citation*** in which it states the sources on which its response is based. The citation effectively makes the chatbot self-classify its responses, and this self-classification is used to automatically decide how to process the chatbot's response.

The flow of generating a response, in broad terms, is as follows:

1. **Generate** a response
2. Extract **commands and arguments** from the response
3. Ensure that the **response is not too long** (generate warning message to chatbot or summarize the response to maintain desired length)
4. Check that the **requested files exist** (if not: generate warnings and regenerate response)
5. Check that the response has a **valid citation** (if not: generate warnings, and, depending on the severity of the error, regenerate the response or proceed to the next filter stage)
6. Overseers (prompted LLM agents) **evaluate** and **refine** the response (if necessary), and return the conversation object along with the validated response and any **feedback** produced in the process of validating the response.

## 1. Syntax that Enables Chatbot to Interact with Backend Scripts

In order to enable the chatbot to request sources in real time as well as enable ways to perform checks of the chatbot's responses, we have defined a syntax and set of conventions for the chatbot to follow in the initial prompt. These conventions enable the chatbot to essentially make commands or produce labels that can be detected and processed automatically by backend scripts. The delimiters of chatbot commands are ‘**¤:**’ and ‘**:¤**’. For example, to request a source on the topic of seeking a diagnosis, the chatbot would generate the message ‘***¤:request_knowledge([“11_seeking_a_diagnosis”]):¤’***. These syntax conventions are described in the chabot’s initial prompt.

## 2. Information Retrieval Algorithm

The strategy behind the Information Retrieval Algorithm is to maximize the proportion of the conversational context that consists of information that is relevant to answering the user’s query. Relevant sources are inserted into the conversation as system messages that are invisible to the user. In order for the chatbot to access documents in its knowledge base its responses are generated in three steps: **1. Perform a Knowledge Request**: The conversational agent requests relevant sources with a knowledge request command (described below), **2. Insert Knowledge**: Insert the relevant sources into the conversation as system messages of the form “*source <source name>: <source content>”* **3. Generate Response**: Allow the LLM to generate a new response which is now informed by the knowledge-enriched context. The chatbot can request at most 2 sources per attempted response.

### 2.1 Requesting Knowledge

In order for the chatbot to request a source, the chatbot has been instructed to request information with messages of the form: **request_knowledge([list of sources])**, e.g.: *request_knowledge([“13_stigma”, “53_psychotherapy”])*. Such messages get interpreted by backend scripts which ensures that the requested sources get inserted into the conversation as system messages, e.g.: “*source 71_health_and_lifestyle_intro: Many people with …*”.

If the chatbot requests a file that does not exist or requests a file that is already inserted into the conversation, corresponding warning messages are inserted into the conversation, and the chatbot is asked to generate a new message after having been warned of its error. The chatbot is instructed to keep making knowledge requests until it is satisfied with the retrieved information (after a source has been inserted, it can proceed to generate a response or make another knowledge request), or until it has made at most 2 requests.

**How does the chatbot know what to request?** In the initial prompt, the chatbot is provided with a list of manually written source summaries, e.g.:

*4. DEALING WITH THE DIAGNOSIS: WHAT NOW?*

*- `41_info_for_newly_diagnosed`:*

*- who to confide in*

*- chances of recovery and improving symptoms*

*- encouraging statistics*

See the Initial Prompt for the full list of summaries.

### 2.2 Citations

The chatbot is instructed to start each message (except for knowledge requests) with a ***citation****,* which is a command of the form **cite([list of references])**, for example ¤:*cite([“13_stigma”]):¤.* The references are used to decide how to process a chatbot response, and provides a way of checking if the chatbot is behaving sensibly. References can be to sources or but can also reference what class of responses the current response belongs to. Other than sources the possible references are

- *initial_prompt*: reference to the initial prompt
- *sources_dont_contain_answer*: used if the chatbot has been unable to find a source that can answer the users question.
- *no_advice_or_claims*: the chatbot is not giving any advice or claims. This is used, for instance, when it is describing its role or informing the user of its limitations.
- *support_phone_number*: the chatbot is referring the user to an emergency phone number (more on this below)

The citations are used to determine the ***mode*** of the chatbot. Currently, there are only 2 modes:

- *source-conveyor mode*: the chatbot is producing an answer based on a source. This label is assigned if the chatbot has cited sources.
- *default mode*: the chatbot has not cited a source.

The mode is used to determine which overseers are called upon in the CAF to evaluate and refine the response (see Section 4), and thus how the response is to be evaluated and processed.

The use of citations is controlled via backend scripts to ensure that the chatbot adheres to the desired conventions. Warning messages are automatically inserted into the conversation if the chatbot

1. Does not start its message with a citation expressed in proper syntax
2. Cites non-existent sources or labels not mentioned in the prompt
3. Cites a source that is not currently available in the conversation

Error 1 and 2 are critical errors; in these cases, the response is deleted, warning messages are inserted, and the chatbot generates a new message which is informed by the warning messages. Error 3 results in a warning message only.

## 3. Referring Unstable Users to Emergency Contact

As the chatbot is interacting with individuals with schizophrenia, it is important to consider how it deals with individuals who are in a state of psychosis, or are in some other unstable psychological state (e.g., suicidal) that requires immediate attention from medical professionals. In such cases, we do NOT want the chatbot to provide guidance, but instead refer them directly to an appropriate emergency contact (currently a dummy number). The instructions associated with these situations are in the initial prompt, and are enforced by the *role_and_emergency_contact* judge (see Section 4).

## 4. Critical Analysis Filter

After a response has been generated it gets passed to the CAF where it is to be evaluated and processed by prompt-engineered LLM agents that are used in the CAF to critically analyze and refine the chatbot response. ***Judges*** are responsible for critically evaluating the chatbot’s responses, and delivering a ***verdict*** like DENY or ACCEPT which determines how the response is to be treated in subsequent parts of the CAF. Each judge is responsible for checking a response against a specific subset of rules, and the judges that get called to evaluate the response are determined by the mode of the chatbot (see 2.2 Citations). The judges currently implemented are:

**Default Mode Judges**

- *disclaimer_check*:
  - This judge determines if the chatbot is unsupported. If unsupported claims are detected, it then assesses if the following criteria are met:
    - Basic and uncontroversial
    - Does not dealing with a sensitive topic, like suicide or depression
      - Includes a cautionary disclaimer of the sort: *”My sources do not answer this question. In general, I do think that …, but you should verify this with a health professional.”*
- *role_and_emergency_contact*:
  - This judge is responsible for ensuring that the chatbot does not start taking on the role of a therapist or some kind of expert (especially since no source has been cited). In addition, it checks if the user is displaying signs of not being in a stable psychological state. If it does detect signs of instability, it checks if the user is referenced to the correct emergency contact number (see Section 2).

**Source-communication Judges**

- source_fidelity:
  - Compares the chatbot's response to the cited sources, and evaluates if the response is consistent with the content of those sources.

The judges are instructed to deliver their analyses in a **format that** **encourages structured reasoning**, as this was found to improve the quality of their reasoning and overall performance. For example, the judge that checks assertions and disclaimers when the chatbot is in default mode may produce the following output:

***user****: "Will going to bed earlier make me sleep better?"*

***chatbot****: "Going to bed earlier can improve sleep quality by aligning with*

*natural circadian rhythms and allowing more restorative deep sleep, leading to*

*increased alertness and well-being."*

***disclaimer_check****:*

*GIVING_ADVICE_OR_CLAIM: "chatbot recommends going to bed earlier and claims it*

*will provide better sleep, so TRUE"*

*SAFE: "The recommendation constitutes a generally accepted view, so TRUE"*

*RELEVANT: "Achieving good sleep is relevant to schizophrenia and mental health, so TRUE"*

*HONEST: "Chatbot does not mention that claim and advice is not based on sources*

*and does not encourage verification by health-care-provider, so FALSE"*

*SAFE_RELEVANT_HONEST: "Criteria HONEST is not satisfied, so DENY."*

In this example, the verdict “DENY” is extracted from the output and used to guide subsequent decisions.

### 4.1 Preliminary and Chief Judges

We observed that GPT-4 was more likely to generate sensible outputs and conclusions that we agreed with, but it would sometimes take over 30 seconds to produce an output, whereas GPT-3.5 rarely took more than 1 second (these values fluctuate considerably, presumably due to server load). We therefore decided to include a preliminary screening step where we use a faster model, and then call on the computationally expensive model to get final verdicts and feedback for the chatbot if the message gets flagged in the preliminary stage. ***Preliminary judges*** are those powered by GPT-3.5 Turbo (16k context window) and ***Chief judges*** *are* powered by GPT-4 (8k context window).

The chatbot response is first evaluated by the preliminary judges. In general, the preliminary judges produce a chain of structured reasoning, followed by a verdict: a key-word which represents the decision recommended by a judge. Possible verdicts may differ between judges, and can be for example REJECT, WARNING, and ACCEPT. Verdicts are mapped to one of two ***decisions:*** ACCEPT or REJECT. and if any of the preliminary judges outputs is mapped to REJECT, then the chief judges (those associated with the mode) are called upon for final verdicts.

The chief judges outputs are mapped to the decision tokens ACCEPT, WARNING, or REJECT. Warning messages (feedback directed at the chatbot) are generated if the decision token is either WARNING or REJECT. If any of the chief judges REJECT the response it gets passed to the response modifier along with the warning messages of the chief judges (see below). Otherwise, the warning messages (if any) are inserted into the conversation history as reminders to the chatbot of how it ought to behave in its next response.

### 3.2 Response Modifiers

If any of the chief judges reaches the verdict of REJECT, then a response modifier (powered by GPT-4), *compliance_enforcer,* is called on to modify the response accordingly. *compliance_enforcer* takes the chatbot response and the feedback from the chief judges as inputs in its prompt-template, and outputs a modified version of the chatbot message that aligns with the prompt instructions and judge feedback. After the modification, the old warning messages are replaced by a new single warning message that summarizes what has happened in the background of the type “your response was modified to comply with the following rules: ….”.

## 5. Managing Message and Conversation Length

Managing the length of the conversation and chatbot responses can be an effective way of getting the LLM to adhere more reliably to instructions.

**Removing unused sources** One way that we manage the conversation length is by removing inserted sources that are not being actively used. A source is defined as *inactive* if it has not been cited in the last two responses. Inactive sources are automatically removed and replaced by a message stating that they have been removed.

**Truncation** The length of the conversation is truncated (starting with oldest messages) whenever the length exceeds 4500 tokens. A message is not removed if it is an active source.

**Response length** The maximum response length, i.e. the *max_tokens* parameter in *openai.ChatCompletion.create()*, is set to 320. However, we do not want this limit to be reached, since the message is simply truncated if exceeded. Furthermore, due to the cognitive impairments of individuals with schizophrenia, we want to limit the amount of information presented per message (that is, chunk the information) so that the user does not get overwhelmed. Two measures are taken to incentivise concise messages and information chunking:

1. We generate warning messages to the chatbot (insert into the chat as system messages) when it exceeds the thresholds 200 and 250 tokens respectively.
2. If the threshold of 300 tokens is exceeded, then the message gets summarized by *message_summarizer*: an LLM agent prompted to summarize long responses.
3. At the start of each source, we include, when relevant, a recommendation on the number of messages over which the source should be presented, e.g. *“[Present over 2 messages or only what is relevant]”*. The information is enclosed between square brackets to indicate that it is “silent” information that is intended only for the chatbot, and should not be communicated to the user.

### Thoughts on Collecting Feedback

Applying this framework to new use cases will likely require a lot of tinkering by domain experts. If this framework is to be practically feasible, it is therefore important to streamline the process of modifying the chatbot and evaluating the effect of those changes. Specifically, it would be useful to develop a user-friendly interface that enables non-programmers to modify the text-based prompts and resources of the chatbot, testing the effect of those changes (for example using automated tests with facilitators to generate large numbers of query-response pairs), storing snapshots of scenarios where the chatbot behaves undesirably, and understanding which prompts to edit to achieve a desired effect or alter a specific behaviour. Lowering the threshold of chatbot development and evaluation this way can facilitate generalizability by accelerating the rate at which diverse data (from more than one person) can be generated, and issues can be detected. Indeed, a bottleneck in our study that halted collection of human feedback and human conversations is the lack of a technical solution for collecting such data from interactions between volunteer clinicians and the chatbot. For example, an online hosted app that lets individuals with access converse with the chatbot and dump feedback and comments by clicking a “Comment and Export”-button.
