# Supplementary material for: Prompt Engineering an Informational Chatbot for Education on Mental Health Using a Multiagent Approach for Enhanced Compliance With Prompt Instructions: Algorithm Development and Validation
Source: JMIR AI. 2025 Mar 26;4:e69820. doi: 10.2196/69820 (PMC11982747; doi:10.2196/69820)
Supplement: Multimedia Appendix 2 [file ai_v4i1e69820_app2.doc]

Note: every level 3 header (e.g. 11_seeking_a_diagnosis) is a source that the chatbot can request. The sources appear in the same order as in the corresponding sections in the GAMIAN manual.

### # 11_seeking_a_diagnosis

[Present over 2 messages or only what is relevant]

If you have experienced hallucinations, delusions, severe paranoia or

disorganized behaviour leading to a crisis, it may be time to think about

seeking help to identify whether or not there is an underlying mental health

condition.

These could be the symptoms of a condition called schizophrenia; however, the

symptoms of many mental illnesses overlap, so your first step towards getting

help should be to consult your doctor. They can advise you on whether it is

necessary for you to see a psychiatrist.

A diagnosis of schizophrenia should only be made by a qualified psychiatrist.

There is currently no test that can be used to show that a person has

schizophrenia so, to make a diagnosis, the psychiatrist will need to talk with

the person and their family or friends about the symptoms they are experiencing.

The psychiatrist will also need to observe the person’s behaviour over several

weeks to identify any signs of the condition.

Early signs of schizophrenia vary from person to person and can be very

difficult to recognize. Depression, compulsive behaviour, decreased motivation

and asocial behaviour may be mistaken for normal changes that take place during

adolescence or linked to drug abuse. As the following figure shows, however,

these symptoms may be early warning signs of schizophrenia [show:

schizophrenia_early_signs.png | contents shown in enumerated list below].

# Schizophrenia early signs

- Changes in mood

- Inability to cry or laugh

- Excessive crying or laughing

- Depression, moodiness

- Sensory changes

- Hearing voices

- Increased sensitivity to noise or light

- Changes in appearance

- Wearing strange clothes

- Poor personal hygiene

- Changes in activity

- Extreme activity or inactivity

- Fatigue

- Changes in sleep patterns

- Problems at school or work

- Difficulty concentrating

- Declining academic performance

- Changes in behaviour

- Prolonged staring

- Using illegal drugs

- Avoiding social situations

### # 12_what_is_schizophrenia

[Present over 2-3 messages or only what is relevant]

If you have noticed the signs of schizophrenia in yourself or received a

diagnosis, one of the first questions you will have is “What is schizophrenia?”

Schizophrenia is a long-term condition that results from changes in the mind and

causes a range of different psychological symptoms.

People with schizophrenia do not choose their illness, any more than people with

a physical illness do. Importantly, there is no known cause – no one is

responsible for a person developing schizophrenia and no one is to blame.

Schizophrenia is a treatable mental illness and many people with the condition

lead completely normal lives; however, it is often sensationalized in the media,

which has led to a range of misconceptions about the nature of the illness.

# Myths and facts

- People with schizophrenia have multiple or split personalities

- People with schizophrenia do not have multiple personalities

- Schizophrenia is caused by bad parenting

- There is no known relationship between parenting style and

schizophrenia, and there is no reason to blame the parents or

family of a person with schizophrenia; however, tension in the

family may add to the stress of having a mental illness

- People with schizophrenia are lazy

- A lack of energy can be a symptom of the illness

- People with schizophrenia are violent and/or dangerous

- People with schizophrenia can act strangely, but are rarely

violent towards others

- What people with schizophrenia see or hear is not real

- What people with schizophrenia experience is very real to them,

no matter how unbelievable or unrealistic others may find it

- People with schizophrenia will never get better

- Symptoms of schizophrenia improve in many people, and some people

recover completely

- Medication for schizophrenia will stop all of the symptoms of

schizophrenia

- Medication can drastically reduce some symptoms of schizophrenia,

but may not prevent them all

- People with schizophrenia cannot work

- Many people with schizophrenia are in employment; doing something

productive can help people to feel better about themselves

- All people with schizophrenia experience the same symptoms

- Symptoms vary widely from person to person; no two people with

schizophrenia will have exactly the same experience

### # 13_stigma

[Present over 2 messages or only what is relevant]

Stigma can refer to the negative perceptions and attitudes surrounding certain

health conditions or diseases. There are a lot of misconceptions surrounding

schizophrenia, which can affect both those with the condition and those who care

for them. Many people believe that a person with schizophrenia will switch

frequently between normal and bizarre behaviour, as if they have multiple

personalities. This promotes a perception that people with schizophrenia are

unpredictable and should be feared.

They may internalize these misconceptions (self-stigmization). It is important

for them and for family members not to allow others’ perceptions to influence

how they feel about that person. It is important that they view their illness as

something they have, and not something that identifies them.

**'I have schizophrenia. I am not schizophrenia. I am not my mental illness. My

illness is a part of me.'** – Jonathan Harnisch, An Alibiography

The name ‘schizophrenia’ is derived from the Greek verb skhizein, ‘to split’,

and phren, denoting ‘soul, spirit or mind’, and originally described a group of

‘schizophrenias’. Although the current language suggests a single, uniform

condition, there is great variety in the severity of illness and types of

symptoms experienced by people with schizophrenia.

There have been many suggestions for alternative names that better describe the

diversity of symptoms apparent in people with schizophrenia. Indeed, a new term,

‘Togo Shitcho Sho’ or ‘integration disorder’, was adopted in Japan in 2002.

Patient groups in the Netherlands are also taking steps towards realizing a name

change in their country. The Dutch patient society, Anoiksis, has suggested a

new name and new concept for schizophrenia, with the aim of reducing the stigma

associated with the condition.

### # 14_who_gets_schizophrenia

Schizophrenia is a common mental illness, affecting about 1 in every 100 people.

Anyone can develop schizophrenia. It is seen in people of all cultures across

the world, and affects equal numbers of men and women. It can also affect people

of almost any age, although the symptoms of schizophrenia usually first appear

when a person is in their late teens or early twenties.

There have been a number of notable people with schizophrenia: John Nash, the

mathematician and Nobel Prize winner; Lionel Aldridge, the Super Bowl-winning

National Football League player; Vincent van Gogh, the artist; and Tom Harrell,

the jazz musician. These examples show that people with schizophrenia can

achieve great things.

### # 21_causes

[Present over 2 messages or only what is relevant]

If you are affected by schizophrenia, you may wonder at some point whether it is

your fault. The answer to that question is no. No one is to blame for

schizophrenia. The reasons why some people develop the condition and some do not

are currently unknown, although there are some factors that are thought to

increase a person’s susceptibility to the condition.

Schizophrenia is sometimes referred to as a heritable condition, which means

that if there are certain genes in a person’s DNA their risk of developing the

illness is increased compared with people without those genes. The chance that a

person will develop schizophrenia is increased from about 1 in 100 to about 1 in

10 if one of their parents has the condition.

In identical twins with exactly the same genetic make-up, however, the chance of

one twin developing schizophrenia if the other twin has the condition is only

50%, and so we know that genetics are not the sole cause of the condition.

Likewise, there is no single `environmental factor` that is known to cause

schizophrenia, although there are certain factors that can increase a person’s

chances of having the illness: these include growing up in a city center,

abusing drugs such as cannabis and amphetamines, and experiencing stressful life

events. There is some evidence that the environment a person is exposed to can

interact with their genes. Research into this phenomenon is ongoing, but not

enough is currently known to say who will or won’t develop schizophrenia.

[show: schizophrenia_genes_environment_interactions.png | relationship between

genes, environment, and illness]

A key feature of schizophrenia is that different combinations of symptoms are

seen both between individuals and within an individual over time – whether this

has anything to do with an individual’s genes and/or environment is still

unclear.

### # 31_symptoms

[Present over 3 messages or only what is relevant]

- “The only thing you have for measuring what’s real is your mind...

so what happens when your mind becomes a pathological liar?” – Neal

Shusterman, Challenger Deep

Schizophrenia affects the way you think, feel and act. Each person

with schizophrenia will have different combinations of symptoms,

making one individual’s experience very different from another’s. It

can be difficult to link the way you are feeling to the way in which

doctors describe the symptoms of schizophrenia. Medical professionals

often divide the symptoms of schizophrenia into three categories:

positive, negative and cognitive. The following pages explain what

these categories mean and give some examples.

# Positive symptoms

This term is used to describe feelings or experiences that do not

correspond to reality, such as hallucinations, delusions or paranoid

thoughts.

- Hallucinations – This means hearing, seeing, feeling, tasting or

smelling something that is not really there. – Hearing voices is a

common hallucination; some people may find the voices non-threatening

or even comforting, while others may find them disturbing and

frightening.

- Delusions – Delusions or deluded thinking means strongly believing

in something that cannot be true. – This can be things like thinking

that you are being watched, believing that you are a famous person,

or believing that the television or radio is sending you signals.

- Paranoid thoughts – Paranoia is extreme distrust, or believing the

worst. – Examples include feeling that other people are plotting

against you or are trying to harm you.

# Negative symptoms

This term describes a lack of feelings or behaviours that are usually

present. Negative symptoms can include low motivation or mood, or

withdrawing from family and friends.

- Low motivation – This can be losing interest in any or all aspects

of life. – A lack of energy can mean that you find it difficult to do

simple things, such as getting out of bed.

- Lack of emotion – This lack of feeling can be shown by a lack of

expression, a flat tone of voice or a lack of eye contact.

- Social withdrawal – This is not wanting to socialize with family or

friends, preferring to spend most of your time alone.

# Cognitive symptoms

This term is used to refer to changes in mental capacity, such as

difficulties concentrating or remembering things.

- Disorganized thoughts and speech – Fragmented thinking can come

across in the way you speak and is characteristic of schizophrenia. –

Examples include responding to questions with an unrelated answer,

and saying illogical things.

- Lack of concentration or attention – A lack of concentration or

attention can mean that tasks like reading a book or watching a

television programme become very difficult and frustrating. – Thought

processes can be slowed, which can make interacting with others

difficult.

- Memory problems – Remembering events or information, or learning

new things, may become challenging.

# A personal perspective on the symptoms of schizophrenia

- “I have felt for a long time, from the beginning of this, that I am

in a different dimension. My feelings and thoughts are not natural.

They are a stranger’s, they are not mine. I have felt that I am

special; that I have discovered the truth, the authority in the

background. I have felt that I am a messenger between the universe

and the earth. I am in a special position; I am the chosen one. I

have felt that everybody wants to hide something from me. They don’t

want me to know the truth. I have felt that everybody is playing a

role. And everybody is talking about me, and they can hear my

thoughts. For a long time I was afraid to go out in the streets. I

didn’t have control of reality. My thinking was not normal. I was

watching the ‘signs’ that ‘they’ were trying to send and show me. I

made connections between my feelings and thoughts and the outside

world. It was scary. I could express these things only after the bad

period: in that state, I could not. It was a changed state of mind.

I had no private life. I thought that the people wanted to

communicate with me about something without speaking.” – István

### # 32_illness_progression

[Present over 3 messages or only what is relevant]

# How will the illness progress?

Once the signs of schizophrenia begin to show, you may notice that

patterns in your condition emerge. Having a different range of

symptoms at different times is to be expected; there are distinct

‘phases’ of schizophrenia, which can occur in cycles.

[show: 32_stages_of_progression.png | show before presenting the 3 stages]

## Prodromal (beginning) phase

- You may notice a gradual change in mood and behaviour before any

obvious and serious changes occur

- You may learn to recognize the early warning signs of a relapse into

the active phase of the illness

## Acute (active) phase

- This is the point when symptoms such as hallucinations, delusions

and disordered thinking become impossible to ignore

- You may reach a crisis point during this phase – if this happens,

the safest place to be may be in hospital

## Residual phase

- Most people find that their condition stabilizes and that severe

symptoms begin to fade away with treatment

- Some symptoms may remain, but this can vary widely from person to

person

- You may experience relapses into the prodromal and acute phases, but

these can be minimized with appropriate medication and support

# Other explanations for schizophrenia-like symptoms

- Substance abuse – Using illegal drugs may cause symptoms of

psychosis including hallucinations or changes in personality.

- Combining medications – Taking medications that interact with each

other may lead to hallucinations.

- Bipolar disorder – In some instances, such as during severe manic

episodes, people with bipolar disorder may experience hallucinations

and delusions.

- Depression – Changes in mood, personality and energy may be signs of

depression. In severe cases of depression, known as psychotic

depression, people may experience delusions.

### # 41_info_for_newly_diagnosed

# Who should I tell?

Who you decide to tell about your illness is completely up to you.

Perhaps a useful comparison is to the situation of having a physical

illness – if you had a physical illness that significantly affected

your life, who would you tell? The answer to this question may give

you an idea of who you should tell about having schizophrenia. It is

often a good idea to explain your condition to the people with whom

you have the closest relationships; for example, those you live with

and those you work closely with. It may not be necessary to tell

people who you do not have strong bonds with. Ultimately, everyone’s

situation is different, and only you will know what is best for your

own situation.

# Will I get better?

[show: statistics_on_who_gets_better.png] 1 in 5 people will recover

completely within 5 years. 3 in 5 people will improve, but may still

experience symptoms occasionally. 1 in 5 people will continue to have

troublesome symptoms.

There is a common misconception that people with schizophrenia have no

chance of recovery. This is not true: the symptoms of schizophrenia

can improve with appropriate treatment, and some people recover

completely. Recovery is a process that involves learning to manage

your symptoms, developing support systems and working towards your

goals. Appropriate ways to manage your condition with medication and

therapy are outlined in the next part of this guide.

### # 51_management_strategy_intro

* “Mental illness is not a choice, but recovery is.” – Unknown

The aims of treatment are to relieve symptoms, prevent psychotic

episodes and enable the person with schizophrenia to progress along

the road to recovery [show: factors_of_recovery.png]. A treatment

plan that combines medication with psychotherapy, often referred to as

‘talking therapy’, and peer-to-peer contact is often the most

effective approach, but everyone is different and it is important to

find the right combination of therapies for you.

### # 52_medication

[Present over 3 messages or only what is relevant]

“I think that medication keeps me going, and it has played a crucial

role until now. My goal is to reduce my medication little by little. I

try to be quite educated – I read a lot about it and I discuss it with

my doctor.” – Monika

# Deciding on a medication

There are many different medication options for schizophrenia and it is

important to find the right one for you [show

medication_effects_side_effects_and_administration.png]. The medication your

psychiatrist recommends will depend on a number of things, including how you

feel about your treatment, how well the medicine controls your symptoms and

whether you suffer any unacceptable unwanted effects.

Different medications reduce some symptoms of schizophrenia more than

others, so you will need to decide which symptoms you most want to

control. Like many medicines, those used to treat schizophrenia can

also have side effects. Different medications will have different side

effects from each other, and the effects of one medication may change

over time. Your psychiatrist will monitor your progress once you have

begun treatment, and may suggest options for reducing side effects

such as adjusting the dose or switching to a different medication.

Another consideration is that medications can be taken in different

ways; for example, medicines can be taken daily as tablets or liquids,

or injected every few weeks.

With so many options and factors to consider, it is important that you

discuss what you most want from your treatment with your psychiatrist

as early on in the process as possible; for example, you may have a

specific aim for your treatment, or there may be a specific symptom or

side effect that you want to avoid. Finding a psychiatrist with whom

you ‘click’ can make talking about your condition easier, and this can

help to ensure that you make decisions about goals for your treatment

together.

“I think that medications have different effects on people with mental

illness. For me, six or seven kinds of antipsychotics were tried over

10 years. Finding the right medication was a turning point in my life.

My basic antipsychotic was an injection, which was given every second

week for 5 years, and I took antidepressants too. In 2011, I felt that

there was no more improvement with this medication and, after

discussion with my doctor, I changed to pills. Since then we have

achieved a big improvement in my health. I am still taking the pills

with an antidepressant. We have reduced the amount of medication since

2011. This has to be done little by little.” – István

# Sticking to medication

Once you start receiving medication, it can be tempting to think that

your lack of symptoms means that you don’t need the medication any

more. If you start skipping doses, however, it is likely that your

symptoms will return; up to 80% of people who stop taking their

medication relapse within 1 year.4 In some instances, suddenly

stopping your medication can be harmful. Therefore you should always

talk with your psychiatrist before making any decisions about your

treatment. Above all, if you decide to stop taking your medication, it

is important that you tell someone.

# Coming of medication

[QUERY FOR RELEVANCE] There may be some special situations when it

might be necessary to stop taking your medication; for example, during

pregnancy or while breastfeeding. In these cases, the benefits to you

of the medication will be assessed against its risks to the baby. You

may be advised to discontinue your medication long enough before you

become pregnant for the medicine to be cleared from your system;

however, this will depend entirely on your individual situation, so it

is crucial that you discuss with your psychiatrist any plans you have

for starting or expanding your family.

“Medication is important because it protects me from having a

psychotic episode. I take my pills every night if I don’t take them I

know that there’s a possibility of a psychotic episode.” – Ronan

### # 53_psychotherapy

[Present over 3 messages or only what is relevant]

There are many different types of psychotherapy but the common goals

that they all share include:

- solving problems that you are currently having

- teaching you skills to overcome problems in the future

- helping you to become self-sufficient.

Psychotherapy usually involves sitting down with a therapist, either

one to one or in a group, and engaging in open dialogue about your

condition.

Discussions of how to cope with your illness may include how to

manage your mood and how to respond positively to whatever triggers

your symptoms. A psychotherapist may sometimes encourage you to

explore difficult and painful emotions and experiences, such as

feelings of anxiety and depression, or specific traumatic events. For

this and other reasons, it is important to find a therapist who you

feel at ease with, to maximize the potential benefits. Indeed, if you

and your therapist trust and respect each other, the treatment is more

likely to work.

A psychotherapist may sometimes encourage you to explore difficult

and painful emotions and experiences, such as feelings of anxiety

and depression, or specific traumatic events. For this and other

reasons, it is important to find a therapist who you feel at ease

with, to maximize the potential benefits. Indeed, if you and your

therapist trust and respect each other, the treatment is more likely

to work.

The specific goal of cognitive behavioural therapy is to change

unhealthy patterns you may have developed in your thinking or

behaviour. A structured approach of identifying goals and setting

small tasks for you to practise between sessions is used to address

specific problems. This approach is also used to teach you the

techniques to manage problems that may arise in the future.

Group therapy describes any psychotherapy in a social setting.

Although group therapy does not necessarily mean talking in a group of

people with schizophrenia, it can have a number of advantages over

other psychotherapies. You may feel more at ease sharing your thoughts

with others who have experienced similar things, as well as a reduced

level of fear of being discredited and a greater level of trust.

Family-focused therapy recognizes that schizophrenia can affect

whole families, not just the person with the condition. The specific

aims of family-focused therapy are to aid communication between family

members and to build a supportive environment for the person with

schizophrenia.

There are many other types of psychotherapy; for example,

hypnotherapy, psychoanalysis, and expressive therapies such as art and

music therapy. Some of these are likely to be more useful to you than

others and, importantly, some may interfere with the management

strategy you and your healthcare team have agreed on. Therefore, you

should talk to your psychiatrists and psychotherapists about any new

approach you may want to try.

Some questions to consider asking your therapist

- What kind of therapy do you offer?

- Do you belong to a professional organization?

- Do you have experience of working with people with schizophrenia?

- How long do the sessions last?

- How often are they held?

- How many sessions am I likely to need?

- Can I contact you between sessions if I need to?

- What sort of results can I expect?

- How long before I should expect to feel some benefit from therapy?

- What does the therapy cost?

### # 54_psychoeducation

Psychoeducation is based on the idea that the more you and your family and

caregivers know about your condition, the better you will be able to manage it

together. It involves a trained therapist delivering a clearly defined programme

that has been designed to educate participants about mental illness. A course of

psychoeducation may include a series of sessions over months or years, and can

be aimed specifically at caregivers and family members, at people with

schizophrenia, or a combination of both.

Because the therapy involves taking in a lot of information, going to sessions

when your condition is most stable is likely to be most effective. Many people

find that they benefit not only from the information they receive during

psychoeducation but also from the learning process itself. Psychoeducation

should be used in combination with psychotherapy and medication, and can help

you develop coping strategies that use your own knowledge of your illness.

Furthermore, it can be a great help to family members and caregivers, as a

better understanding of the condition can help them to recognize and meet the

needs of the person with schizophrenia.

### # 55_support_groups

Living with schizophrenia can be isolating at times. Joining a support group can

be a good way to socialize and share experiences with others who know what it is

like to live with the condition. Many support groups have been set up by people

with schizophrenia, and include people who are successfully managing their

condition and moving on with their lives.

Different groups specialize in different types of support, and can operate at

local, national and international levels. One benefit of joining a support group

is that it can be much easier to talk about your problems and feelings with

people who know what you are going through than with, for example, a family

member or therapist. People with schizophrenia are experts in their own

condition, and so they may be able to give helpful advice based on their

experiences. Sharing stories and finding common ground can also create a bond of

friendship.

[source 101_further_information has references to support groups and other

resources]

### # 56_reluctance_to_seek_help

[Present over 2 messages or only what is relevant]

There may be times when a family member or friend thinks that you should seek

medical help, but you believe that you are well and feel reluctant to do so.

Talking to someone who has been in a similar position may be useful, as they may

help to ease your fears.

As a family member or friend, it can be extremely difficult to broach this

subject, and the way that you react can be important in determining the outcome

for the person with schizophrenia. First, it is a good idea to discuss the

situation together as early as possible, or at a time when the person with

schizophrenia feels well; the more time that passes, the more likely it is that

a crisis point will be reached. A support group for family members and

caregivers may be able to provide valuable advice.

Secondly, try speaking to someone who can give you the perspective of a person

with schizophrenia. If possible, try to arrange for a doctor to visit the person

with schizophrenia at home. This may be particularly useful as it can allow all

members of the household to be involved.

If, as a person with schizophrenia, you do not accept help, it is important for

you to know that there is a legal process that can lead to your compulsory

admission to hospital and treatment if there is a risk of harm to you or others.

This would be a very distressing course of action for all involved and so family

members and caregivers should see this option as a last resort. Further

information on compulsory hospital admission and treatment will be available

from your local support group, citizen’s rights organization or the healthcare

service in your country.

### # 61_personal_relationships

[Present over 2 messages or only what is relevant]

Schizophrenia is not easy to live with. It can put strain on your relationships

with people in your family or those you share a home with. During relapses in

your illness, you may not realize the demands that are being made of people

around you; when you emerge into recovery, you may find yourself feeling rather

lonely. Likewise, it can be difficult to live with a person with schizophrenia.

Nevertheless, there are some practical steps you can take together to make

things easier.

[Present only if relevant] If you are planning a family, there are a number of

issues that you need to discuss with your partner early on, such as the risk of

your child developing schizophrenia, how you will manage having a child, and, if

you are a woman, the possibility that you will relapse after giving birth or

during the pregnancy. There will be decisions that are not easy to make, but

information from your healthcare team can aid in difficult discussions. As

mentioned in Chapter 5, some medications used to treat schizophrenia may not be

suitable for use during pregnancy. Therefore, you should discuss your options

with your doctor before trying to conceive a child.

[present `S` to schizophrenia patients and `F` for family members (ask if

unsure)]

- Learn more about schizophrenia

- S: Explain it to your friends and family

- F: Try to be understanding of your family member’s behaviour

- Be honest

- S: Explain it to your friends and family

- F: For example, about your fears and your hopes

- Agree on boundaries

- S: Tell people close to you what you want them to do and,

importantly, what you don’t want them to do

- F: Respect your family member’s wishes as far as possible

- Build trust

- S: Trust that your family want what is best for you

- F: Show the person that you have their best interests at heart

- Don’t be ashamed

- S: You are not to blame for your illness and it is nothing to be

ashamed of

- F: There is nothing shameful about caring for someone with

schizophrenia

- Learn from others

- S and F: Support groups will have members who have been in similar

situations and can help

### # 62_work_relationships

Many people with schizophrenia are able to continue working once their

condition is under control. Others decide to adjust their work

commitments to suit their condition. This can include changing to a

less stressful role, reducing their number of working hours or taking

up a job share.

Some employers are more flexible than others in their attitudes

towards allowing people to change their work contracts; however,

national and international laws are in place to protect against

discrimination in the workplace. If you need to find out more about

your rights, there are a number of sources of help and guidance

available to you, including the human resources department of your

company, citizen’s rights organizations and schizophrenia support

groups.

A personal perspective on working as a person with schizophrenia “I’m

a mechanical engineer by first degree, and I also studied business

administration. I design machine parts for a big company in Israel. I

do have good colleagues at work, and I appreciate them very much. I

consider it a good place to work. My colleagues at work notice when

I’m getting better. They don’t bother me too much, and they don’t ask

difficult questions – they let me continue with my day without

sticking their noses in things that are a bit delicate.” – Ronan

### # 63_social_life

It can be difficult to maintain a social life if you have

schizophrenia. As mentioned earlier in this guide, schizophrenia can

lead to symptoms including being less able to socialize and a lack of

emotion, so you may lose touch with people you were friends with. As

you start to recover, however, you may find yourself wanting to go out

and socialize more.

Support groups can be a good way to meet and socialize with other

people with schizophrenia, but it is important not to surround

yourself completely with others who have the condition. Meeting and

socializing with people without the illness can help you to see a

world outside of schizophrenia. Psychotherapy can help you to regain

your confidence and overcome the social barriers associated with

mental illness, to enable you to have a normal social life. Caring for

someone with schizophrenia can also affect your social life; it is

important to recognize this and to take steps to make sure your own

needs are met. Sharing responsibilities with other family members

and/or caregivers can be a good way to allow you to have some time to

yourself, to socialize and to do other things that you enjoy.

### # 71_health_and_lifestyle_intro

Many people with schizophrenia struggle with their physical health as well as

their mental health. They have a high risk of heart disease and obesity, which

means that people with schizophrenia have a shorter life expectancy than the

general population. One reason for this could be that the symptoms of

schizophrenia make it difficult to maintain a healthy lifestyle; some unhealthy

behaviours, such as eating unhealthy foods, drinking alcohol, taking illegal

drugs and smoking, are common in people with schizophrenia.

Furthermore, some of the medications used to treat people with schizophrenia can

have a negative effect on general health; for example, weight gain is a common

side effect of many medicines for the condition. Whether or not you choose to

adjust your lifestyle is up to you, but there are several ways in which looking

after your general health can help in the overall management of your condition.

### # 72_diet_exercise_sleep_alcohol

[Present over 2 messages or only what is relevant]

[show: 72_exercise_diet_alcohol_influence.png | illustrates effect of

lifestyle-factors]

Some treatments for schizophrenia can increase the risk of weight gain and

obesity. Sticking to a healthy diet is a good way to minimize this risk. Your

doctor will monitor your weight and, if weight gain becomes a problem, it may be

worth discussing different medication options.

Drinking to excess should be avoided, not only to improve your general health

but also because there is a strong association between schizophrenia and

alcoholism, which means that the risk of becoming addicted to alcohol is greater

if you have schizophrenia. Therefore, it is a good idea to moderate your

drinking as much as possible.

There are several reasons to incorporate a physical exercise regimen into your

lifestyle. First, getting a good amount of exercise will help you to control

your weight in combination with a healthy diet. Secondly, exercise can put you

in a better frame of mind, as hormones released during exercise are associated

with improved mood. Exercise can also improve the way you sleep.

People with schizophrenia tend to have disrupted sleep patterns: you may find

yourself sleeping more or less than most people, or at different times of the

day. This can lead to excessive tiredness, known as fatigue. A change in sleep

patterns can be the first sign of a psychotic episode, and therefore can be

taken as a warning sign. Maintaining a regular routine can help you to get your

sleep patterns back to normal. This can include having a bedtime routine, going

to bed at the same time and waking up at the same time. Relaxation techniques,

such as meditation, can help to reduce stress; this is important because stress

can stop you sleeping peacefully. Caffeine should be avoided, especially in the

evening, as it can disrupt your sleep.

[`sleep_assistant` provides in-depth info on sleep if user is interested]

### # 73_hygiene

Schizophrenia can change the way that you care for yourself: poor personal

hygiene is common among people with the condition. This aspect of the illness

may arise from symptoms such as a lack of motivation or delusions. Dental

problems in particular can develop due to dry mouth caused by medication, or if

you forget to take care of your teeth. Therefore, it is important to tell your

dentist what medication you are taking and to follow their advice on oral

health. Making a checklist of daily tasks, such as brushing your teeth,

showering and getting dressed, can help you to avoid self-neglect. Doing these

tasks at the same time each day can help to establish a daily routine.

### # 74_sex

Healthy sexual relationships are important and you should not ignore this part

of your life. Schizophrenia itself and some medications for schizophrenia can

lead to reduced sexual desire. On the other hand, you may experience unusual or

strong sexual feelings. Increased sexual behaviour and strange thoughts of a

sexual nature are likely to be related to the changes in the mind that occur as

part of the condition. As schizophrenia usually first becomes apparent during a

person’s late teens and early twenties, when libido is typically high, it is not

surprising that changes in sexual thoughts often occur at this time. If you have

a tendency to take part in high-risk sexual behaviour, your risk of getting a

sexually transmitted disease, such as HIV, is high. Therefore, it is important

to understand the risks and to seek advice for maintaining good sexual health

from your doctor.

### # 81_living_with_schizophrenia_intro

“I want a good relationship with my wife, with my children, with my family and

with my colleagues at work. I don’t want to have another psychotic episode, but,

if it happens, I know I can manage and people around me know how to deal with

it.” – Ronan

As you begin to recover from your illness, it is likely that you will want to

start regaining control of your life. This can include learning how to avoid

triggering a relapse, and developing coping strategies for when times get tough.

# Warning signs of a relapse

- Moodiness, aggression or depression

- Excessive crying or laughing

- Hallucinations or talking to oneself

- Changes in activity or sleep patterns

- Changes in behaviour

- Being asocial

- Making meaningless statements

- Strange postures or prolonged staring

- Changes in appearance or poor personal hygiene

There are several things that are thought to increase a person’s chances of

relapsing into the acute phase of schizophrenia, such as high stress levels,

stressful life events and the use of illegal drugs.

### # 82_stress

Stress is a common trigger for schizophrenia and, unfortunately, it is extremely

difficult to avoid stress completely because having schizophrenia can be

extremely stressful in itself. Nevertheless, it is possible to manage your

stress levels and to reduce the impact that stress has on your life.

[show: 82_how_to_avoid_stress.png | illustrates points below]

- Learn which situations you find stressful and avoid them

- Talk to someone – a problem shared is a problem halved

- Avoid alcohol, caffeine and illicit drugs, as these can cause

anxiety

- Learn some relaxation techniques

- Simplify your life - just do one thing at a time

- Stick to a schedule for each day

- Exercise - this can prevent as well as relieve stress

- Withdraw gradually from situations you find stressful

[`relaxation` assistant provides in-depth info if user is interested | Not

implemented, but might be implemented in the future]

### # 83_illegal_drugs

It is common, especially for young people, to turn to drugs such as cannabis or

amphetamines to try and block out the symptoms of schizophrenia. You may think

that illegal drugs will help to numb feelings of depression or anxiety but,

although some drugs may give temporary relief, in the long term they may

actually make symptoms of schizophrenia worse or cause a relapse.

Some illegal drugs can increase symptoms such as paranoia, hallucinations and

delusions. As the effects of illegal drugs may worsen the symptoms of

schizophrenia, it can be difficult for your doctors to be sure that the

medication you are receiving is working properly if you are taking other drugs

at the same time. More importantly, mixing drugs can be highly dangerous to your

health.

### # 84_depression_and_suicidality

[Present over 2 messages or only what is relevant]

# Depression and suicidality

About one in three people with schizophrenia will experience low mood

or depression at some point. Tragically, suicide rates are higher in

people with schizophrenia than in the general population. Therefore,

it is of the utmost importance that you recognize the warning signs of

suicidality and learn ways in which to combat negative thoughts.

# Signs of suicidality

- Feeling like a burden to family and friends Feeling hopeless or

helpless, as if there is no solution to the problem at hand

- Talking about wanting to die

- Reckless or dangerous behaviour

- No longer fearing death

- Drinking excessively or abusing drugs

- Asocial behaviour

- Writing a suicide note

Although at the time it may seem like you will feel this way forever,

there are several things that you can do to beat depression, and it is

crucial that you talk to someone about your feelings.

ways to improve your mood

- Talk to your psychiatrist: you may be suffering from a depressive

illness that is treatable.

- Get some rest: a good night’s sleep can make you feel a lot better

and your outlook may be different in the morning.

- Get a change of scenery: you may associate one particular place with

being depressed, so leaving this place can reduce these feelings.

- Visit friends or family: isolation and boredom can worsen negative

feelings, so socializing with others is a good way to prevent this.

- Do something you enjoy: don’t forget that there are things that make

you happy.

- Learn to relax: ask your doctor or therapist about relaxation

therapies, such as meditation.

- Talk to someone: sharing your problems with others, whoever they may

be, can be a weight off your mind.

- Phone a helpline: helplines can be useful when you need to talk but

feel you have no one to talk to. If you feel you are close to a

crisis, it is of the utmost importance that you tell someone –

people who work for helplines can give you the practical advice and

support you need.

### # 91_advance_directive

One way of making sure that the decisions made in your life are your own, even

during times of crisis, is to have a plan of how you would like to be treated if

you do lose control of your behaviour. You can record any wishes you might have

in an ‘advance directive’ [show: 91_advance_directive_card.png]. [Notify user

that future versions of this chatbot might have a dedicated chatbot agent for

helping to create an advance directive card]

The advance directive may include the name and contact details of a family

member or caregiver who is to be given authority to act as an advocate on your

behalf, as well as information relating to your preferences on medication,

finance and housing.

### # 92_legal_rights

Advice on your legal rights can be obtained from your healthcare team, citizen’s

rights organizations and support groups. There are two reasons why it may be a

good idea to investigate your rights. First, if your condition places

limitations on your ability to work, you may be entitled to financial benefits.

Secondly, it is a good idea to know your legal rights if abnormal behaviour

during psychotic episodes leads to dealings with the police and legal system.

### # 101_further_information

[Present over 2 messages or only what is relevant]

# Networking and other sources of information

## International organizations

### EUFAMI

European Federation of Associations of Families of People with Mental Illness

The European representative organization for family support

associations across Europe. EUFAMI promotes the interests and

wellbeing of families and caregivers affected by severe mental

illness. http://www.eufami.org/

## GAMIAN-Europe

Global Alliance of Mental Illness Advocacy Networks-Europe

A patient-driven, pan-European organization that represents the

interests of people affected by mental illness and advocates for their

rights. http://www.gamian.eu/

## ISF

International Schizophrenia Foundation

A non-profit organization with international affiliates dedicated to

raising the levels of diagnosis, treatment and prevention of the

schizophrenias and allied disorders.

[`Inform that the website is currently unavailable`]

## WFMH

World Federation for Mental Health

An international organization for the promotion of mental health.

http://wfmh.com/

## Patient groups, support groups and other useful websites

### Intervoice

The International Hearing Voices Network

A network of people who hear voices, see visions or have other unusual

perceptions. http://www.intervoiceonline.org/

## Mind

A charity that provides advice and support to empower anyone

experiencing a mental health problem.

https://www.mind.org.uk/information-support/types-of-mental-health-problems/schizophrenia/about-schizophrenia/

## Rethink Mental Illness

A charity working to help everyone affected by severe mental illness

to recover a better quality of life.

https://www.rethink.org/advice-and-information/about-mental-illness/learn-more-about-conditions/schizophrenia/

# Further reading

Anoiksis. Psychosis? Me? Anoiksis, 2014. Available from:

https://www.gamian.eu/activities/archive/message-ge-members/

[If they are curious: this booklet has been written by and for people

who are susceptible to psychosis. This is the updated link]
